# Ticking Down Sodium Levels—An Atypical Link Between Chronic Hyponatremia and Borreliosis

**DOI:** 10.3390/biology14040427

**Published:** 2025-04-16

**Authors:** Raluca Maria Vlad, Carmen Vasile, Alexandra Mirică

**Affiliations:** 1 Department of Paediatrics, “Carol Davila” University of Medicine and Pharmacy, 050474 Bucharest, Romania; raluca.vlad@umfcd.ro (R.M.V.); alexandra.mirica@umfcd.ro (A.M.); 2 “Grigore Alexandrescu” Emergency Children’s Hospital, 011743 Bucharest, Romania

**Keywords:** SIADH, borrelia, Lyme disease, chronic hyponatremia, serum osmolarity

## Abstract

Lyme disease (LD), caused by the spirochete *Borrelia burgdorferi*, is the most prevalent tick-borne disease in Europe, including Romania, where endemic areas are well documented. It has a wide range of clinical manifestations and severity, including rare neurological complications. Persistent hyponatremia is an atypical presentation of Lyme neuroborreliosis and can be associated with the syndrome of inappropriate antidiuretic hormone secretion (SIADH). SIADH is characterized by unregulated antidiuretic hormone release, leading to impaired water excretion, dilutional hyponatremia, and low serum osmolality. We report the case of a 16-year-old female with clinically well-tolerated, but severe, refractory hyponatremia, who was poorly responsive to intravenous sodium supplementation and fluid management. Complex investigations ruled out multiple causes of hyponatremia; neuroborreliosis was confirmed via positive *Borrelia* serologies, despite the absence of a suggestive history of exposure. SIADH likely symptomatology resulted from central nervous system inflammation induced by *Borrelia*, a mechanism rarely documented in the medical literature. Treatment with antibiotics and fluid restriction led to a gradual improvement in fluid balance and sodium homeostasis. This case emphasizes the importance of considering rare infectious causes, such as LD, in patients with unexplained SIADH, especially in endemic areas. It highlights the importance of a multidisciplinary approach in intricate, complex cases.

## Simple Summary

Lyme disease (LD), caused by the spirochete *Borrelia burgdorferi*, is prevalent in Europe, particularly in Romania, and can lead to a wide range of symptoms, including nervous system involvement. One extremely rare complication of this disease might be persistent hyponatremia, which can be associated with the syndrome of inappropriate antidiuretic hormone secretion (SIADH). In this study, the authors report the case of a 16-year-old girl who developed severe, chronic hyponatremia which was later found to be linked to LD. The few cases published suggest that LD may cause this condition through inflammation in the nervous system. The clinical case presented aims to highlight the potential causative link between LD and SIADH, underlining the importance of differential diagnosis and a multidisciplinary approach in complex, intricate cases. The authors’ clinical experience among very few others may help raise suspicion, improve understanding, and help clinicians reach an early diagnosis and ensure that patients receive early treatment in similar cases in the future.

## 1. Background and Aim

### 1.1. Introduction

Lyme disease (LD) is an infectious disease caused by the spirochete bacteria *Borrelia Burgdorferi*. Romania is an endemic country in Europe, as evidenced by the 532 confirmed human LD cases it reported in 2018 [1]. 

Affecting multiple systems and organs, LD presents with a wide range of clinical manifestations and severity, correlated with the stage of the illness. The disease progresses through three distinct stages: early localized phase, early disseminated phase, and late phase, each characterized by specific clinical manifestations [2,3]. In the early stage, several days or weeks post-tick bite (typically within a range of 3 to 30 days), 60–80% of infected patients develop skin lesions [2].

**Figure 1:**
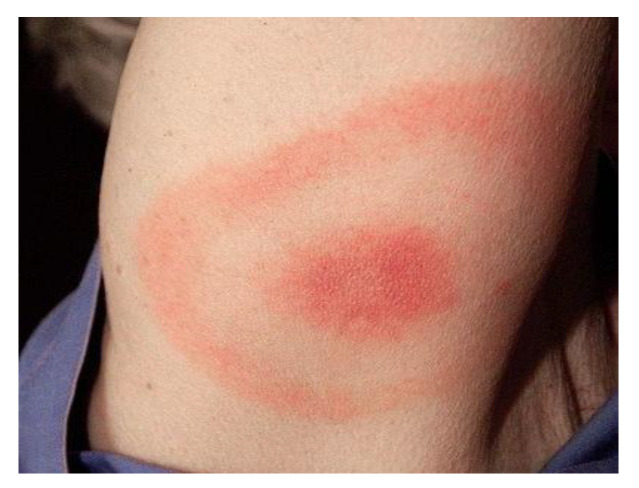
Erythema migrans. Close-up image of a circular, expanding rash with target-like appearance [6].

The primary manifestation of early LD is erythema migrans (EM), a rash that emerges at the site of the tick bite, usually within 7 to 14 days following the bite. Over the course of days or weeks, the rash may spread circumferentially, often exhibiting central clearing, which leads to a characteristic “target” or “bull’s-eye” appearance [2,4]. Early infection may be asymptomatic or cause nonspecific symptoms such as influenza-like symptoms, fever, lethargy, headaches, and muscle or joint pain [5]. Figure 1 depicts the typical EM of LD [6].

The early disseminated phase usually occurs several weeks or months after a tick bite (regardless of whether EM has been present before). It may be the first manifestation of LD. This stage is characterized by neurological, cardiac, and ocular manifestations and cutaneous findings [5].

Neuroborreliosis, observed in 10–15% of infected individuals in both Europe and North America, typically manifests as meningoradiculitis, meningitis, or meningoencephalitis. The classic triad of acute neurologic findings includes meningitis, cranial neuropathy, and motor or sensory radiculoneuropathy. Additional manifestations may include carditis, conjunctivitis, keratitis, iridocyclitis, retinal vasculitis, choroiditis, optic neuropathy, uveitis, borrelial lymphocytoma, and multiple EMs [5,7,8].

**Figure 2:**
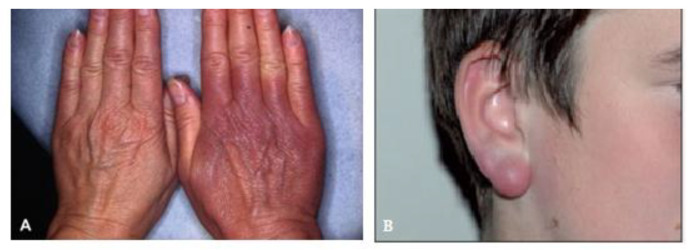
Skin manifestations of *Borrelia* infection: (**A**) acrodermatitis chronica atrophicans-unilateral violet discoloration on the extensor surface of the hand [10]; (**B**) borrelial lymphocytoma—bluish-red nodule on the ear lobe [11].

In the later stages of *Borrelia* infection, which may manifest months or even years post-infection, clinical features can include skin manifestations like acrodermatitis chronica atrophicans (Figure 2A); borrelial lymphocytoma (Figure 2B); intermittent or persistent chronic arthritis (less common in Europe; most common in the United States); and rare neurological complications such as encephalomyelitis or chronic neuroborreliosis [9].

Severe and chronic hyponatremia is an atypical feature of Lyme neuroborreliosis, posing a diagnostic challenge to attribute it to the syndrome of inappropriate antidiuretic hormone secretion (SIADH) [12]. SIADH may be misdiagnosed due to the uncommon relationship between severe and refractory hyponatremia and Lyme neuroborreliosis [13].

SIADH is a condition characterized by the uncontrolled release of antidiuretic hormone (ADH) from the pituitary gland or ectopic sources, or by its persistent action on vasopressin receptors. Impaired water excretion in SIADH causes hyponatremia with either hypervolemia or euvolemia. Most commonly, SIADH occurs as a secondary condition due to an underlying disease [10,11,14,15,16].

CNS infections, such as LD, may trigger an inflammatory cascade with the elevation of pro-inflammatory cytokines like interleukin-6. This inflammation response can disrupt normal physiological processes, leading to altered brain function and various systemic effects. In the case of SIADH, the inflammation may stimulate the enhanced secretion of ADH, further exacerbating electrolyte imbalances. SIADH can also be caused by a CNS illness through the “reset osmostat” mechanism. In this process, the brain adjusts its regulatory set point for osmolality, resulting in the retention of water and a reduction in serum sodium levels due to an increased release of ADH [10,13].

### 1.2. Aim

The authors aim to highlight the potential causative link between SIADH and LD, underlining the importance of differential diagnosis and a multidisciplinary approach in complex, intricate cases. The authors’ experience among very few others may help raise suspicion, improve understanding, and help clinicians reach an early diagnosis and ensure that patients receive early treatment in similar cases in the future.

## 2. Materials and Methods

We present a rare case of neuroborreliosis manifesting as SIADH in a nearly asymptomatic teenager with chronic severe hyponatremia. The patient was admitted to the Pediatrics Department at Grigore Alexandrescu Emergency Clinical Hospital, Bucharest, Romania, in July 2024. Written consent for publication was obtained from the patient’s father.

## 3. Case Presentation

A 16-year-old adolescent female was transferred from an Infectious Diseases Clinic to our department for the evaluation and management of severe hyponatremia refractory to intravenous rehydration with sodium-rich solutions. She presented persistent nausea, vomiting, and slight deterioration of her general condition.

The patient reported a two-week record of asthenia, fatigue, and muscle weakness. Three days before admission, she developed afebrile, bilious vomiting (2–3 episodes/day) without other symptoms. Admitted to the Infectious Diseases Clinic, acute hepatitis was ruled out and she was transferred to our department for further investigations. From the patient’s medical history: two weeks before admission, she was treated for an Escherichia coli urinary tract infection with a 5-day course of cefuroxime, and one month before admission, she had an episode of lipothymia (hypotonia, marked fatigue, and dizziness) with spontaneous recovery.

Upon admission, pulmonary, cardiovascular, renal, and abdominal examinations were normal, except for a low appetite and a vomiting episode 12 h prior. The patient did not show evidence of edema, nor clinical signs of virilization. She had a slender figure with a weight of 45 kg, a height of 166 cm, and a BMI of 16.3 kg/m^2^. Details regarding the onset of secondary sexual characteristics were unavailable due to the patient’s mother’s death when she was 9 years old, and her father could not provide all these data. She had regular menstrual cycles (30-day intervals) without clinically significant premenstrual syndrome.

Laboratory investigations (as illustrated in Table 1) revealed severe hyponatremia (persistently low values ranging from 112 to 118 mmol/L), hypochloremia, hypomagnesemia, normal serum potassium and calcium levels, no metabolic acidosis, and normal lactate levels. Serum osmolality was reduced. Renal function tests were normal. Vitamin D deficiency was identified. She had slightly decreased urine density, normal urinary ionogram and creatinine, and urinary osmolality. Hormonal investigations were within the normal limits, including estradiol, FSH, LH, testosterone, dehydroepiandrosterone sulfate (DHEAS), 17-OH progesterone, deoxycortisol, morning cortisol, and ACTH. Serum aldosterone was normal, with mildly reduced plasma renin activity. The sweat test results were normal.

The abdominal ultrasound findings were within the normal limits.

Various differential diagnoses associated with chronic, severe, and persistent hyponatremia were considered. We excluded diabetes mellitus due to the absence of polyuria, polydipsia, consistently normal glucose levels, and a lack of metabolic acidosis. We also ruled out chronic kidney disease and renal tubular acidosis due to there being no relevant history, no edematous syndrome, normal blood pressure in both supine and standing positions, normal renal function tests, and normal arterial blood gas findings. Cystic fibrosis was considered due to the patient’s low BMI, below the second percentile, but it was unlikely without a history of chronic respiratory disease or chronic diarrhea with steatorrhea. She also had normal sweat test results.

We took into consideration anorexia nervosa with the potential abuse of diuretics/laxatives. However, repeated anamnesis (from the adolescent and her father), a psychological assessment, and psychiatric evaluation did not indicate any signs of body image distortion, abnormal eating behavior, or chronic medication intake. Given the impossibility of continuous monitoring, this hypothesis remained unlikely. The patient denied excessive water intake (potomania). The urinary ions count did not show increased sodium excretion. The hospital’s psychologist described the patient as having perfectionist tendencies, high academic performance, and introverted traits. The patient denied abnormal eating behavior, although this could not exclude an eating disorder. The psychiatrist evaluated the patient as having an anxious temperament, academic perfectionism, cognitive rigidity, and chronic difficulties regarding social interactions. Both the patient and her father denied concerns related to diet or body image. She was diagnosed with anxiety disorder and social communication disorder, but an eating disorder was ruled out as a possible cause of persistent hyponatremia.

The endocrine evaluation revealed normal thyroid function, negative Anti-Thyroid Peroxidase Antibodies (ATPOs) and Anti-Thyroglobuline (ATGL), and normal basal cortisol, ACTH, and DHEAS levels. The gonadal axis evaluation indicated low–normal estradiol and normal gonadotropins and prolactin, with the aldosterone and renin results being within the normal limits. The patient denied headaches, polyuria, polydipsia, or cardiovascular issues. Additionally, the clinical assessment identified a slender build, mildly dry skin, absent active stretch marks, minimal subcutaneous fat, Tanner stage B5P5, and mild periareolar hirsutism.

We excluded a range of endocrine disorders that could justify chronic, severe hyponatremia: for 17/21 hydroxylase deficiency, there were no clinical criteria (no signs of virilization), DHEAS was within the normal limits, 17-OH progesterone and deoxycortisol were normal; for hypoaldosteronism, blood pressure remained normal, there were no changes in serum potassium, the urinary ionogram was normal, and serum aldosterone and plasma renin activity were within the normal limits; for adrenal insufficiency, serum cortisol and ACTH were normal; there were no clinical signs of severe myxedema or fT4; and TSH and anti-thyroid antibodies were normal. In addition, to exclude cortisol hypersecretion, we performed a cortisol plasma rhythm at 23 h, which showed normal values.

**Figure 3:**
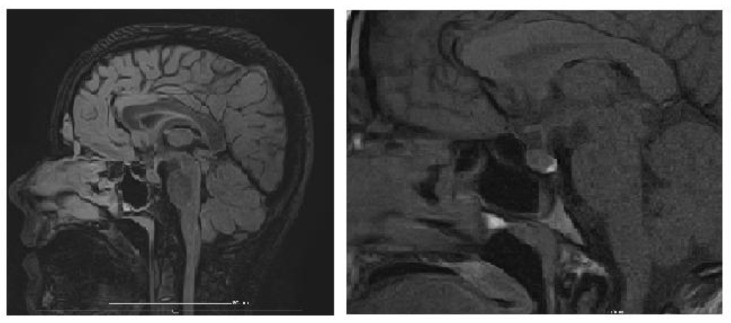
Brain MRI (dynamic post-contrast sequence) showing a 3 × 2 mm pituitary microadenoma.

An MRI of the brain and pituitary gland was performed, revealing a pituitary microadenoma (Figure 3), most likely an incidental finding, ruling out other CNS pathologies that would explain chronic hyponatremia (tumor, trauma, vascular acquired conditions, or malformations). The dynamic post-contrast sequence shows a small 3 × 2 mm area with delayed enhancement in the adenohypophysis, considered unrelated to the patient’s symptoms, as pituitary hormone levels were normal, suggesting an incidentaloma.

Diabetes insipidus/ADH overproduction was also considered but remained unlikely: the patient did not exhibit polyuria/oliguria, and both urinary density and ionogram were normal. The patient remained euvolemic (clinically and paraclinically) throughout hospitalization, with low serum osmolarity. Urinary osmolarity increased with sodium infusion and/or water restriction.

During hospitalization, the patient received 80 mmol of sodium per day through rehydration therapy and oral sodium supplements, maintaining sodium levels within the same range. Clinically, she had a good general condition, with alleviated nausea, normal blood pressure, present diuresis, intact sensorium, and no paroxysmal manifestations.

An intravenous sodium loading attempt was made: for 72 h, the patient received 1000 mL of saline and 1000 mL of glucose with electrolytes, along with oral salt supplements. Under this regimen, serum sodium rose to 127 mmol/L, serum osmolarity increased to 265 mOsm/kg, serum potassium remained within the normal limits, the arterial blood gas test was normal, and the urinary ionogram showed a slight increase in urinary sodium, with a moderate rise in urinary osmolarity (as illustrated in Table 1). The patient initially received sodium through rehydration therapy and oral supplements, which maintained sodium levels within the same range. Following this, an intravenous sodium loading attempt was made, leading to a slight increase in serum sodium, serum osmolarity, and urinary parameters.

**Figure 4:**
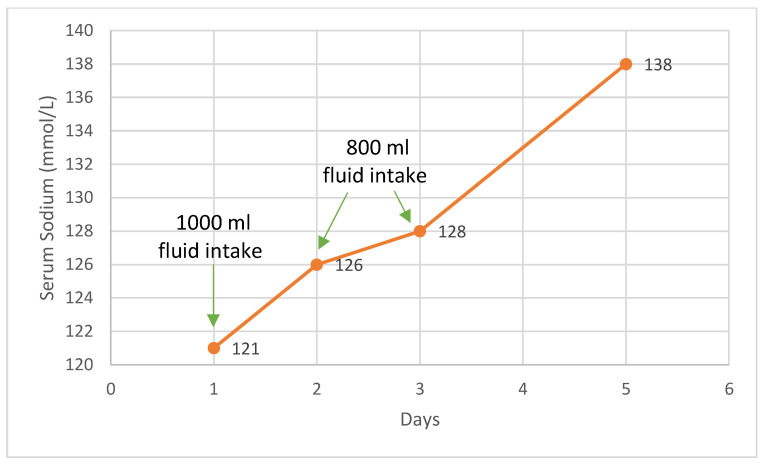
Serum sodium evolution during fluid restriction. Note the gradual increase in plasma sodium levels over the four days following the initiation of fluid restriction to 800 mL per day, without additional intravenous sodium.

At this point, SIADH was taken into consideration as the underlying cause of the patient’s hyponatremia. The patient presented with severe, chronic hyponatremia that was poorly responsive to intravenous supplementation, well tolerated clinically, with euvolemia, normal blood pressure, low serum osmolarity, and increased urinary osmolarity with sodium supplementation. Copeptin levels were slightly increased. We initiated a therapeutic trial, limiting the fluid intake to 800 mL per day, without additional intravenous sodium, which led to favorable outcomes, including the normalization of sodium levels and an increase in serum osmolarity over the next four days. The fluid restriction was implemented, resulting in the gradual normalization of plasma sodium levels. The patient responded positively to fluid restriction (Figure 4). Follow-up assessments at 1 month and 6 months post-treatment indicated a steady improvement in fluid tolerance and plasma sodium levels.

Despite extensive investigations, the etiology of SIADH remained unclear. An infectious, traumatic, or vascular cause of CNS pathology has been excluded both clinically and through MRI. A computed tomography (CT) scan of the thorax and abdomen did not reveal any tumors. Concerning a potential infectious etiology, laboratory testing ruled out *Brucella* spp., *Epstein–Barr virus*, *Cytomegalovirus*, *human Immunodeficiency virus*, *hepatitis B virus*, and *hepatitis C virus*, as well as *herpes simplex virus*, *Toxoplasma gondii*, *Toxocara* spp., and *Mycobacterium tuberculosis.*

Given the positive therapeutic response (normalization of sodium levels after fluid restriction), the diagnosis of SIADH was set. The copeptin (ADH) level was 11 pmol/L, with a serum osmolality of 270–280 mOsm/kg, falling within the reference range (<11.60 pmol/L for 270–280 mOsm/kg).

The patient was also evaluated in the pediatric nephrology department, where a form of nephrogenic syndrome of inappropriate antidiuresis (NSIAD) was considered but deemed unlikely. This condition can occur due to an activating mutation of AVPR2, a pathology typically affecting males and associated with very low serum levels of arginine vasopressin (AVP). However, in the patient’s case, AVP was inappropriately elevated, and therefore genetic testing and initiation of therapy with Tolvaptan were not justified.

We received positive Western blot and IgG for *Borrelia*. The infectious etiology of SIADH associated with LD was considered, and antibiotic treatment with Cefaclor was initiated for 3 weeks. She was also advised to maintain a fluid restriction at home, limiting intake to 1500 mL/day and a high-protein diet.

One month after completing the antibiotic treatment, she returned for follow-up investigations to assess the possibility of gradually increasing her fluid intake. She reported a fluid intake of 1500 mL per 24 h at home. Upon admission, her serum sodium and urinary ionogram were normal, and the calculated serum osmolarity was 292 mOsm/kg (within normal limits). The fluid intake was increased to 1600–1800 mL/24 h, with oliguria of approximately 600 mL/24 h, cloudy, slightly hyperchromic urine with minimal foam. Sodium decreased to 127 mmol/L, chloride decreased to 97 mmol/L, potassium remained normal, and urinary sodium and chloride were normal, while urinary potassium was low (16 mmol/24 h). Serum osmolarity decreased to 271 mOsm/kg. Consequently, fluid intake was reduced to 1500 mL/24 h, and urine output increased to approximately 900 mL/24 h. Sodium and chloride in plasma continued to drop, suggesting persistent SIADH; therefore, maintaining the fluid restriction (1000–1500 mL/24 h) is recommended (as illustrated in Table 1). During this hospital stay, the patient presented with normal blood pressure (values between 90/60 and 105/80 mmHg), without edema, and reported dizziness following increased fluid intake and decreased sodium levels.

Six months post-antibiotic therapy, she returned for follow-up investigations to reassess the possibility of gradually increasing her fluid intake. At home, her fluid intake ranged from 1000 to 1300 mL per 24 h. On admission, she was completely asymptomatic, her sodium levels were normal (136 mmol/L), urinary sodium slightly increased (270 mmol/24 h), urinary chloride slightly increased (265 mmol/24 h), and the calculated serum osmolarity (286 mOsm/kg) was normal.

During this second fluid trial, fluid intake was increased to 1900 mL in the first 24 h, with a diuresis of approximately 1000 mL/24 h, and 1700 mL in the following 24 h, with a diuresis of 900 mL/24 h. After 48 h of fluid loading, the patient reported fatigue, nausea, and dizziness. Blood tests revealed a decrease in sodium (136 mmol/L to 125 mmol/L), serum chloride (103 mmol/L to 93 mmol/L), and calculated serum osmolarity (286 mOsm/kg to 261 mOsm/kg), measured osmolarity (282 mOsm/kg to 259 mOsm/kg). Potassium levels remained normal, while urinary sodium (270 mmol/24 h) and chloride (265 mmol/24 h) were slightly elevated, with normal urinary potassium levels. Western blot for *Borrelia* was still positive; therefore, another course of antibiotics was recommended (Doxycycline) and fluid restriction was maintained, with the patient now tolerating 1500 mL/day.

## 4. Discussion

Hyponatremia is among the most common electrolyte abnormalities in children. It is characterized by plasma sodium levels <135 mmol/L. Sodium homeostasis is crucial for maintaining intravascular volume and is tightly regulated by ADH and the thirst mechanism [17]. Clinical manifestations of hyponatremia typically appear when sodium levels drop below 125 mEq/L and may include confusion, headaches, lethargy, muscle cramps, and reduced reflexes. More severe symptoms, such as seizures, coma, brain herniation, or death, are associated with cerebral edema [18]. The adolescent admitted to our service for the evaluation and management of severe hyponatremia refractory to intravenous hydration with hypertonic solutions reported a two-week history of asthenia and muscle weakness, followed by several episodes of vomiting. Despite very low sodium levels, she had a good general condition: she was euvolemic with normal blood pressure, no edema, and no other specific findings. No paroxysmal episodes were reported. We concluded that the sodium levels decreased slowly over a long period, resulting in mild/nonspecific symptoms.

The etiology of hyponatremia is diverse, and accurate diagnosis requires understanding its pathophysiology. A thorough history, physical exam, and basic lab tests, including serum osmolality and tonicity assessment, are essential. Normal serum osmolality ranges from 285 to 295 mOsm/kg. Sodium is the primary determinant of serum osmolality [19]. Tonicity refers to the concentration of particles capable of exerting osmotic force across a membrane and is determined by solutes that cannot freely cross, such as sodium. Urea and similar particles are ineffective in this regard [20]. Hyponatremia can be associated with isotonic, hypertonic, or hypotonic serum conditions, with hypotonic hyponatremia being the most common, characterized by reduced serum osmolality (<275 mmol/L). It is classified based on extracellular volume status: normovolemic, hypervolemic, or hypovolemic [19].

Euvolemic hyponatremia, often seen in SIADH, is caused by excessive ADH secretion, leading to volume expansion and increased natriuresis due to natriuretic peptides [19].

A multidisciplinary team, including a pediatrician, endocrinologist, nephrologist, psychiatrist, and psychologist, systematically ruled out multiple diagnoses through extensive clinical and paraclinical investigations. The conditions excluded comprised diabetes mellitus, renal tubular acidosis, cystic fibrosis, anorexia nervosa, diuretic and laxative abuse, NSIAD, nephrogenic diabetes insipidus, and various endocrinopathies, such as 17α-hydroxylase and 21-hydroxylase deficiencies, hypoaldosteronism, adrenal insufficiency, and hypothyroidism.

SIADH is characterized by the unregulated release of antidiuretic hormone from the pituitary gland or ectopic sources or its persistent action on vasopressin receptors. First identified in 1967 by William Schwartz and Frederic Bartter in two patients with lung cancer, the diagnostic criteria they established remain the standard today. SIADH results in impaired water excretion, leading to hyponatremia associated with either euvolemia or hypervolemia [21,22,23].

The clinical manifestations of SIADH arise from hyponatremia and reduced extracellular fluid osmolality, which promote water movement into cells and cerebral edema. Symptoms depend on the severity and rapidity of sodium decline, along with the extent of cerebral edema. Early signs of acute hyponatremia, which typically occur when serum sodium levels drop below 125–130 mEq/L (normal: 135–145 mEq/L), include nausea and malaise. Vomiting is often a forewarning of more severe complications. A more pronounced and acute decrease in sodium levels can lead to headache, lethargy, seizures, coma, and respiratory arrest, especially when levels fall below 115–120 mEq/L [24].

In chronic hyponatremia, the brain undergoes adaptive mechanisms, allowing patients to remain asymptomatic even with sodium concentrations below 120 mEq/L. However, nonspecific symptoms such as nausea, vomiting, gait disturbances, cognitive and memory impairments, fatigue, dizziness, confusion, and muscle cramps may occur. These subtle signs are often overlooked during clinical evaluations. Approximately one-third of patients with chronic hyponatremia and sodium levels below 120 mEq/L experience nausea and vomiting [24]. This is further supported by our patient’s minimal symptoms, with clinically well-tolerated hyponatremia [24].

On physical examination, patients with SIADH are typically euvolemic, exhibiting normal skin turgor, blood pressure, and moist mucous membranes without signs of jugular venous distension or peripheral edema. A comprehensive neurological and pulmonary assessment is imperative for identifying underlying causes.

There is no definitive test for diagnosing SIADH. Patients typically present with hyponatremia and an apparent euvolemic state. In 1967, Schwartz and Bartter proposed diagnostic criteria that remain valid today [11,25]. Table 2 presents the criteria proposed by Schwartz and Bartter alongside the corresponding values for our patient. Our patient met all the criteria proposed by Schwartz and Bartter, regarding serum sodium concentration, serum osmolality, urine sodium concentration, and urine osmolality, confirming the diagnosis.

She exhibited no signs of volume depletion, with normal skin turgor and blood pressure. Other potential causes of hyponatremia were excluded, including adrenal insufficiency, hypothyroidism, cardiac failure, pituitary insufficiency, renal salt-wasting conditions, hepatic dysfunction, and medications affecting renal water excretion. Additionally, pseudo hyponatremia causes, such as hyperglycemia and hyperuricemia, were ruled out, as the patient demonstrated normal glucose levels and renal function tests. Implementing a fluid restriction of 800 mL per day, without additional intravenous sodium, resulted in favorable outcomes, including the normalization of serum sodium levels and an increase in serum osmolality.

In SIADH, a combination of ADH-induced water retention and secondary solute loss is observed. Chronic SIADH typically shows a predominance of solute loss over water retention. Treatment focuses on correcting and maintaining sodium levels above 130 mEq/L while addressing underlying conditions [26].

Several indirect modalities are effective in managing SIADH. Fluid restriction remains a cornerstone, aiming to achieve a negative fluid balance by limiting daily water intake (oral, intravenous, and metabolic production) below daily water losses (skin, respiratory tract, stool, urine). This approach increases the serum sodium concentration [26]. In certain cases, hypertonic saline (3% NaCl) is utilized in hospitalized patients to address hyponatremia temporarily. Despite the kidney’s inability to produce urinary sodium concentrations as high as 3% saline (>400 mmol/L), this intervention can transiently raise serum sodium levels [26].

During hospitalization, several therapeutic approaches were implemented while closely monitoring serum and urinary sodium levels, as well as serum and urinary osmolalities (as illustrated in Table 1). The patient initially received 80 mmol of sodium per day through rehydration therapy and oral sodium supplements, which maintained serum sodium levels at 117 mmol/L without significant change. Subsequently, an intravenous sodium-loading protocol was initiated. Over 72 h, the patient was administered 1000 mL of saline and 1000 mL of glucose with electrolytes, alongside continued oral sodium supplementation. Under this regimen, serum sodium levels increased to 127 mmol/L, serum osmolality rose to 265 mOsm/kg, serum potassium remained within normal limits, and arterial blood gas analysis was normal. Additionally, the urinary ionogram revealed a slight increase in urinary sodium levels and a moderate rise in urinary osmolality. Despite the observed increase in serum sodium from 117 mmol/L to 127 mmol/L, the response was less pronounced than expected following saline administration. A therapeutic trial of fluid restriction to 800 mL per day, without additional intravenous sodium, was implemented, resulting in favorable outcomes, including the normalization of sodium levels and an increase in serum osmolality over the next four days. The patient exhibited a good general condition, with alleviated nausea, normal blood pressure, present diuresis, intact sensorium, and no paroxysmal manifestations.

Addressing the underlying cause of SIADH remains critical for correcting hyponatremia and achieving long-term resolution. It can result from various etiologies, including malignancies (most commonly small cell lung cancer), medications (such as carbamazepine, oxcarbazepine, chlorpropamide, cyclophosphamide, and selective serotonin reuptake inhibitors), and less frequently non-steroid anti-inflammatory drugs (NSAIDs), opioids, interferons, methotrexate, vincristine, vinblastine, ciprofloxacin, haloperidol, and high-dose imatinib. Other causes include surgical procedures, hormone deficiencies (hypopituitarism, hypothyroidism), hormone treatments (vasopressin, desmopressin, oxytocin), HIV, pulmonary conditions (e.g., pneumonia), and central nervous system disorders (CNS) (stroke, hemorrhage, infections, trauma, mental health disorders, and psychosis) [10,11,14,15,16].

The association between hyponatremia and CNS infections is well established, with several theories proposed regarding the underlying mechanisms [27]. In some cases, hyponatremia may result from direct electrolyte loss due to fever or vomiting, or the use of hypo-osmolar fluids for rehydration, rather than from SIADH. Additionally, non-osmotic stimuli for ADH release, such as diarrhea, vomiting, excessive sweating, systemic vasodilation, and fluid leakage from the intravascular compartment, are frequently observed in infections and can exacerbate serum sodium depletion [17].

CNS infections that induce SIADH may do so through a “reset osmostat” mechanism where the brain adjusts its osmostat to maintain serum sodium at lower concentrations than normal, facilitated by enhanced ADH release. The reset osmostat phenomenon has been observed in diseases such as tuberculosis and malaria. In 1995, Patwari et al. studied 60 children with bacterial meningitis and found that approximately 35% were diagnosed with SIADH, which correlated with severe meningeal inflammation [28]. The relationship between intracranial inflammation and ADH secretion may be mediated by increased levels of pro-inflammatory cytokines (e.g., IL-6, IL-1β), along with neutrophils, C-reactive protein, and brain natriuretic peptide, all contributing to heightened ADH release. A small study in 1994 involving six cancer patients demonstrated that plasma ADH levels rose in all patients two hours after IL-6 injection, suggesting that this inflammatory cytokine may directly induce SIADH. When inflammatory processes are responsible for SIADH, the syndrome typically resolves following the appropriate treatment of the underlying infection [17].

Regarding the etiology of SIADH, through clinical, paraclinical, and imaging investigations, we have excluded an infectious, traumatic, or vascular cause of CNS pathology both clinically and through MRI. A CT scan did not reveal any tumors at the thoracic and abdominal levels.

Concerning a potential infectious etiology, laboratory testing ruled out *Brucella* spp., *Epstein–Barr virus*, *Cytomegalovirus*, *human immunodeficiency virus*, *hepatitis B virus*, and *hepatitis C virus*, as well as *herpes simplex virus*, *Toxoplasma gondii*, *Toxocara* spp., and *Mycobacterium tuberculosis*.

Lyme disease, caused by the spirochete *Borrelia burgdorferi*, is the most prevalent arthropod-borne disease, predominantly diagnosed in the Northern Hemisphere’s temperate regions, with an estimated 65,500 cases reported annually across Europe [3,13,29]. The most prevalent questing tick species found in Romania’s natural and urban environments is Ixodes ricinus, the castor bean tick, which is also the most common tick species documented in Europe [30]. After the spirochete is introduced into the human host, the disease progresses through three distinct clinical stages: early localized phase, early disseminated phase, and late phase [31]. The initial presentation often includes a characteristic skin lesion, EM, at the tick bite site. With hematogenous spread, the disease can lead to neurologic, cardiac, or rheumatologic complications. Neurologic manifestations occur in approximately 15% of untreated *B. burgdorferi* infections [31].

Chronic refractory hyponatremia is an extremely rare manifestation of Lyme neuroborreliosis [12].

Lyme neuroborreliosis may induce SIADH-like symptoms through CNS inflammation, triggering excessive ADH release. Another mechanism involves a “reset osmostat”, where the brain lowers its sodium set point by enhancing ADH secretion [12]. The association between hyponatremia and neuroborreliosis is notably rare, with only a limited number of cases documented in the clinical literature. In 2004, Shamin et al. described two cases of neuroborreliosis presenting with mild hyponatremia, neuropsychiatric symptoms, and acute demyelinating polyneuropathy involving autonomic dysfunction. In these instances, hyponatremia was mild and effectively resolved with fluid restriction and antibiotic therapy [32]. Similarly, Siddiqui et al. reported a patient whose hyponatremia was successfully managed with fluid restriction and oral sodium chloride supplementation [17]. Recently, Da Porto et al. reported a case of SIADH-induced hyponatremia that was refractory to correction with hypertonic saline infusion but responsive to antibiotic treatment for *Borrelia* [12]. Considering the patient’s symptoms—generalized fatigue, malaise, and myalgia—along with the neurological tropism of Lyme disease and the patient’s origin from an endemic area, we performed serological testing and Western blot for *Borrelia*, which returned positive for IgG and Western blot.

The infectious etiology of SIADH associated with Lyme disease was considered, and antibiotic treatment with Cefaclor was initiated for 3 weeks, in addition to fluid restriction at home, limiting intake to 1500 mL/day. After completing the first course of antibiotic treatment, the patient returned for clinical and paraclinical reevaluation at 1 month and 6 months, attempting to increase fluid intake. We observed a minimal gradual improvement in fluid balance and sodium homeostasis. However, serum sodium levels and plasma osmolarity remained low, along with the onset of symptoms such as fatigue, nausea, dizziness, and low blood pressure. Subsequently, a follow-up Western blot test for *Borrelia* came back positive, leading to the initiation of a new course of antibiotic therapy with Doxycycline and continued fluid restriction.

## 5. Conclusions

The intricate and complex diagnosis of SIADH in children is challenging in the clinical setting. Schwartz and Bartter’s criteria are effective and helpful, but clinicians must remain vigilant to rare etiologies, which can obscure the classic presentation. A thorough workup, including clinical history, laboratory investigations, and imaging studies, is essential for accurate diagnosis and appropriate management.

The management of SIADH, particularly with fluid restriction, underscores the importance of this intervention in normalizing serum sodium levels and improving patient outcomes. While intravenous sodium therapy may be used temporarily, fluid restriction remains the cornerstone of treatment, allowing for a negative fluid balance and promoting the gradual correction of hyponatremia and long-term maintenance of normal serum sodium. The patient’s response to a restricted fluid regimen also demonstrates the effectiveness of simple, non-invasive measures in managing SIADH, even in complex cases.

This case presents a rare cause for chronic, well-tolerated hyponatremia, emphasizing the potential for central nervous system infections to trigger SIADH. The identification of *Borrelia burgdorferi* as the causative agent through serological testing, along with the successful resolution of hyponatremia with complete antibiotic therapy, highlights the importance of considering rare infections in the differential diagnosis of SIADH and particularly Lyme disease in endemic areas.

## Figures and Tables

**Table 1:** Dynamics of laboratory findings over 2 weeks of admission and several trials of fluid and electrolyte management.

	Upon Admission	Rehydration and Oral Sodium (80 mmol/d)	IV Sodium Loading Day 1	IV Sodium Loading Day 3	Fluid Restriction (800 mL/d) Day 1	Fluid Restriction (800 mL/d) Day 5	One-Month Follow-Up	Fluid Intake (1800–1500 mL/d) Day 3	Six-Month Follow-Up	Fluid Intake (1900–1700 mL/d) Day 2
**pH**	7.36	7.36	7.37	-	7.41	7.35	7.34	7.37	7.38	-
**Lactate**	1.13	1.98	1.29	-	1.10	1.82	3.04	1.25	1.39	-
**HCO_3_**	22.8	23	22.1	-	23.2	24.7	26.2	22.6	26.2	-
**Na**	111	117	122	127	121	138	136	124	136	125
**K**	3.97	4.4	4.23	3.92	4.87	4.47	4.62	4.11	4.52	4.61
**Cl**	82	103	91	94	90	102	100	93	103	93
**Ca**	8.8	10.4	-	-	-	-	9.90	8.8	9.7	9.2
**Mg**	1.71	2.17	-	-	-	-	2.06	1.89	2.04	-
**Diuresis (mL)**	-	1500		1800	700	-	600	900	1000	900
**Fluid intake (mL)**	-	600	1000	700	1000	-	1500	1500	1300	1700
**BP**	99/61	106/64	101/54	120/75	103/63	-	90/60	100/80	99/53	107/81
**Urea**	-	34	13	15	24	-	41	26	27	17
**Creatinine**	-	0.42		0.63	0.59	-	0.73	0.51	0.63	-
**Uric acid**	-			2.36	2.69	-	4.58	3.39	4.39	-
**Blood Glucose**	-	115	89	100	-	-	91	89	83	83
**Calculated Serum Osmolarity**	-	253	254	264	-	-	292	262	286	261
**Measured Serum Osmolarity**	-	-	-	-	-	286	289	-	282	259
**Urine Specific Gravity**	-	-	-	-	-	-	-	-	1.019	-
**Urinary Electrolytes** **(mEq/L)**	-	-	-	Na: 147 K: 27 Cl: 157	-	-	Na 250 K 42 Cl 217	Na 201 K 26.4 Cl 199	Na 270 K39.29Cl 265	-
**Urine Osmolarity**	-	-	-	435	-	-	-	-	820	681

**Table 2:** Schwartz and Bartter criteria, alongside case study criteria.

Schwartz and Bartter Criteria	Diagnostic Value	Case Study Value
Serum sodium concentration	<135 mEq/L	121 mEq/L
Serum osmolality	<275 mOsm/kg	248 mOsm/kg
Urine sodium concentration	>40 mEq/L	98 mEq/L
Urine osmolality	>100 mOsm/kg	534 mOsm/kg
Clinical evidence of volume depletion	no	no
Other causes of hyponatremia	no	no
Resolution of hyponatremia through fluid restriction	yes	yes

## Data Availability

The original contributions presented in this study are included in the article; further inquiries can be directed to the corresponding author.

## Abbreviations

The following abbreviations are used in this manuscript:

CNS	Central nervous system
SIADH	Syndrome of inappropriate antidiuretic hormone secretion
NSIAD	Nephrogenic syndrome of inappropriate antidiuresis
ADH	Antidiuretic hormone
AVP	Arginine vasopressin
EM	Erythema migrans
DHEAS	Dehydroepiandrosterone sulfate
ATPOs	Anti-Thyroid Peroxidase Antibodies
ATGL	Anti-Thyroglobulin
NSAIDs	Non-steroid anti-inflammatory drugs
